# Bacteremia Caused by Both *Legionella pneumophila* Serogroup 2 and *Helicobacter cinaedi*

**DOI:** 10.31662/jmaj.2021-0016

**Published:** 2021-07-06

**Authors:** Taiga Nagase, Sachie Wada, Takayuki Yokozawa, Akira Fujita, Toshimi Oda

**Affiliations:** 1 Department of Infectious Diseases, Showa General Hospital, Tokyo, Japan; 2 Department of Hematology, Showa General Hospital, Tokyo, Japan; 3 Department of Clinical Laboratory, Showa General Hospital, Tokyo, Japan; 4 Department of Infection Control and Prevention, Showa General Hospital, Tokyo, Japan

**Keywords:** *Legionella pneumophila*, *Helicobacter cinaedi*, pneumonia, bacteremia

## Abstract

A 74-year-old woman with a history of pure red cell aplasia and hypogammaglobulinemia developed pneumonia. A urine antigen test and sputum subculture on buffered charcoal yeast extract (BCYE)α agar were positive for *Legionella pneumophila*. Serological testing identified *L. pneumophila* serogroup 2. An aerobic blood culture also became positive on day 5; its subculture on BCYEα agar revealed the same pathogen, but that on blood agar revealed *Helicobacter cinaedi*. We thus diagnosed her with bacteremia caused by both pathogens. Hence, in cases of *H. cinaedi* bacteremia along with pneumonia, the screening of other pathogens including *L. pneumophila* is needed.

## Introduction

*Legionella pneumophila* is a pathogen responsible for severe community-acquired or nosocomial pneumonia, especially among immunocompromised hosts or the elderly. It was first reported at the American Legion Convention in Philadelphia in 1976 ^(1), (2)^. *L. pneumophila* serogroup 1 is responsible for >80% of *L. pneumophila* pneumonia ^(3)^. A previous report showed that two of 127 isolates of *L. pneumophila* were of serogroup 2 ^(4)^. Regarding toxicity, there are no reports that serogroup 2 is more invasive than serogroup 1.

*Helicobacter cinaedi*, a Gram-negative spiral-shaped bacillus found in the gastrointestinal tract of humans and other animals, was first reported in 1985 in a patient infected with the human immunodeficiency virus ^(5)^. Bacteremia caused by *H. cinaedi* is reported frequently in immunocompromised hosts ^(6)^. However, reports on bacteremia caused by *H. cinaedi* or other pathogens are rare. To our knowledge, there is no report of bacteremia caused simultaneously by *L. pneumophila* and *H. cinaedi*. Here, we report the first such case.

## Case Report

**Figure 1.**
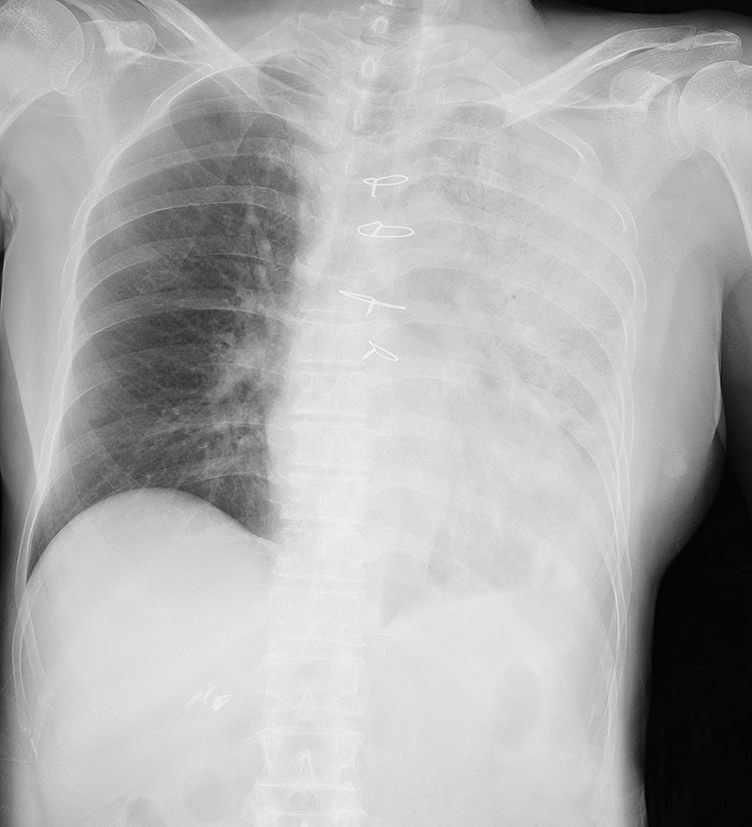
Chest radiography on admission revealed pneumonia over the left lung.

**Figure 2.**
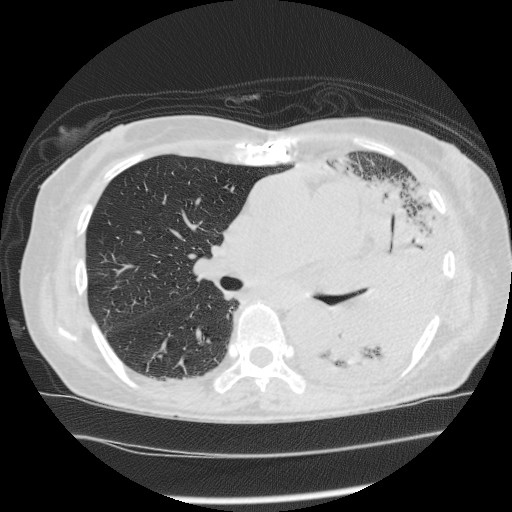
Chest computed tomography on admission revealed consolidation and ground-glass opacity around the left lung.

A 74-year-old woman was admitted to our emergency department with a history of pure red cell aplasia after thymectomy treated with cyclosporin and hypogammaglobulinemia treated with the regular administration of a gamma globulin preparation. Her chief complaint was wandering, which lasted for 3 days. At admission, she was alert, her respiratory rate was 24/min, blood pressure was 47/36 mmHg, heart rate was 186 bpm, and oxygen saturation was 92% with 8 L/min of oxygen. Rhonchi were heard in both lungs, and her tongue was dry and atrophic. The white blood cell count was 7.9 × 10^9^/L, with neutrophil predominance (70%). Her hemoglobin level was 8.0 g/dL, serum urea nitrogen was 55.0 mg/dL, serum creatinine was 3.42 mg/dL, and C-reactive protein was 33.38 mg/dL (Table 1). Chest radiography revealed a decrease in permeability over her left lung (Figure 1), and chest computed tomography revealed infiltration with an air bronchogram over her left lung and a little pleural effusion bilaterally (Figure 2). She previously smoked a quarter pack per day for 5 years in her youth, but had no history of drinking. Twelve days before admission, she visited a hot spring. She had not used a humidifier or circulating bath. No other *L. pneumophila* infections were reported from that hot spring.

**Table 1.** Laboratory Data on Admission (Day 1).

Hematology			Biochemistry			Coagulation		
Hb	8.0	g/dL	TP	4.9	g/dL	PT-INR	1.43	
RBC	252×10^4^	/μL	Alb	2.5	g/dL	APTT	31.6	s
Ht	25.5	%	T-Bil	0.8	mg/dL			
PLT	33.4×10^4^	/μL	ALP	191	U/L			
WBC	7,900	/μL	AST	30	U/L	**Arterial blood gas analysis**		
Neut	70	%	ALT	17	U/L	O_2_ 8 L/minute reserver mask		
Eos	0	%	LD	470	U/L	pH	7.319	
Bas	1	%	CK	280	U/L	pCO_2_	20.5	mmHg
Mon	9	%	Na	133	mEq/L	HCO_3_^-^	10.2	mmol/L
Lym	18	%	K	3.9	mEq/L	pO_2_	85.4	mmHg
RET	11	‰	Cl	97	mEq/L	Lac	8.1	mmol/L
			BUN	55.0	mg/dL			
			CRE	3.42	mg/dL			
			CRP	33.38	mg/dL			

Based on clinical symptoms and imaging findings, pneumonia was suspected. We thus collected samples for sputum culture, blood culture, and urine antigen tests for *Streptococcus pneumoniae* and *L. pneumophila*. As the urine antigen test for *L. pneumophila* (Ribotest^Ⓡ^Legionella, Asahi Kasei Pharma Co., Tokyo, Japan) was positive, we diagnosed her with severe *L. pneumophila* pneumonia and began levofloxacin administration.

*L. pneumophila* was also found in sputum subculture on buffered charcoal yeast extract (BCYE)α agar under microaerobic conditions at 35°C, and slide agglutination tests with monovalent antisera identified *L. pneumophila* serogroup 2. An aerobic bottle of blood culture became positive on day 5, and subculture was performed on blood agar, chocolate agar, and BCYEα agar. Subculture on BCYEα agar revealed *L. pneumophila* under microaerobic conditions at 35°C, and slide agglutination tests with monovalent antisera identified *L. pneumophila* serogroup 2, whereas that on blood agar revealed *H. cinaedi* under the same conditions. We thus diagnosed her with bacteremia caused by both pathogens. We did not perform drug susceptibility tests for these pathogens and did not check for any mutations and toxicity changes.

**Figure 3.**
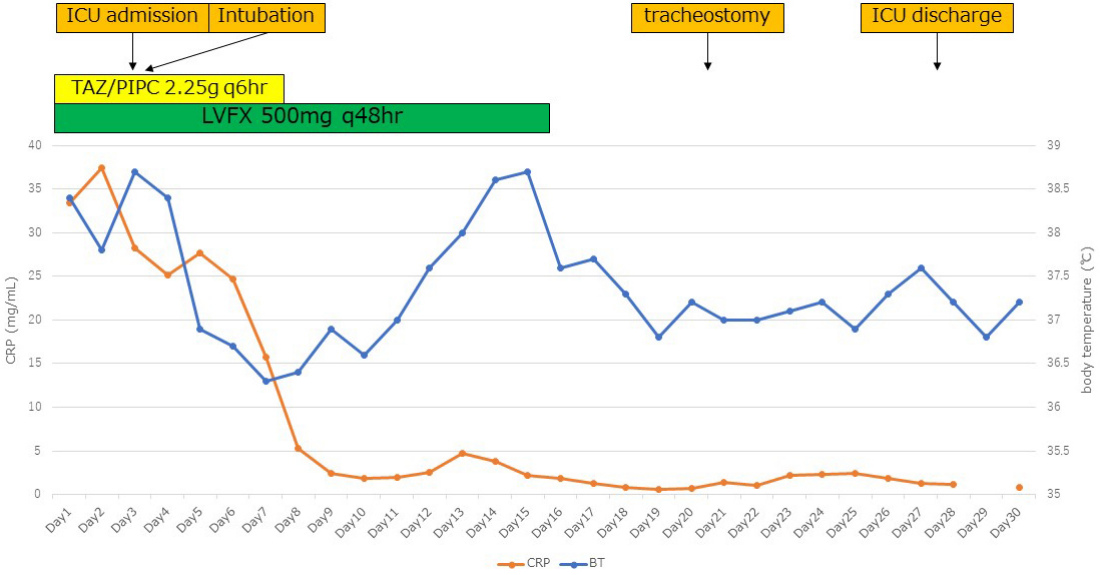
Clinical course of this patient in the acute stage.

**Figure 4.**
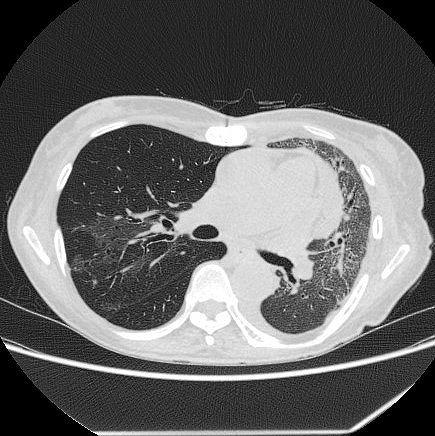
Chest computed tomography before discharge showed that consolidation and ground-glass opacity were improved.

The patient showed disturbances in consciousness, hypotension, and deoxygenation on day 2. She was intubated and transferred to the intensive care unit. Levofloxacin and tazobactam/piperacillin were administered with intensive care. She recovered gradually and was discharged from the hospital on day 60 (Figure 3 and 4).

## Discussion

In *L. pneumophila* pneumonia, the sensitivity of sputum culture is low ^(7), (8)^. Since 2019, a new serological test (Ribotest^Ⓡ^ Legionella) has been available in Japan with greater sensitivity for antigen detection in the urine. In some cases, *L. pneumophila* is not detected in sputum cultures and only urine tests for *L. pneumophila* are positive ^(9), (10)^. Although we cannot identify the serogroup in these cases, blood subculture on BCYEα agar might prove helpful and might make the diagnosis of *L. pneumophila* pneumonia more reliable. As *L. pneumophila* sometimes causes outbreaks, serogroup detection can contribute to epidemiological studies or infection control.

As *L. pneumophila* bacteremia is often reported among immunocompromised patients ^(9)^, blood subculture on BCYEα agar might be effective for detection. Some studies have reported the identification of *L. pneumophila* from a lung biopsy or bronchoalveolar lavage ^(9)^. Although the sensitivity of these tests might be high ^(7)^, these represent invasive techniques. In cases of pneumonia with a positive urine antigen test for *L. pneumophila*, blood subculture might allow for the identification *L. pneumophila* using a less invasive method.

Table 1 shows that the patient’s renal functions were decreased on admission. We assumed that prerenal renal dysfunction was caused by dehydration and renal dysfunction was caused by the extra-pulmonary symptoms of *L. pneumophila* pneumonia. We performed renal replacement therapy for several days, and her renal functions improved gradually.

It is possible that *H. cinaedi* was found as contamination. However, as she developed continuous bacteremia after discharge, it is more likely that bacterial translocation from her intestine occurred. In this case, *H. cinaedi* was the most probable cause of the positive signal in the automated blood culture machine. Without the urine antigen test for *L. pneumophila*, the possibility of *L. pneumophila* bacteremia would have been overlooked. To our knowledge, there is no report of *H. cinaedi* causing pneumonia. Thus, in cases of *H. cinaedi* bacteremia with pneumonia, the presence of other pathogens including *L. pneumophila* needs to be examined.

## Article Information

### Conflicts of Interest

None

### Author Contributions

All authors were involved in data interpretation. All authors critically revised the report, commented on drafts of the manuscript, and approved the final report.

### Informed Consent

All study participants provided informed consent.
